# MultiSourcDSim: an integrated approach for exploring disease similarity

**DOI:** 10.1186/s12911-019-0968-8

**Published:** 2019-12-19

**Authors:** Lei Deng, Danyi Ye, Junmin Zhao, Jingpu Zhang

**Affiliations:** 1 School of Computer Science and Engineering, Central South University, Changsha, 410075 China; 2 School of Computer and Data Science, Henan University of Urban Construction, Pingdingshan, 467000 China

**Keywords:** Disease similarity network, Diffusion component analysis, Integrating multiple data sources

## Abstract

**Background:**

A collection of disease-associated data contributes to study the association between diseases. Discovering closely related diseases plays a crucial role in revealing their common pathogenic mechanisms. This might further imply treatment that can be appropriated from one disease to another. During the past decades, a number of approaches for calculating disease similarity have been developed. However, most of them are designed to take advantage of single or few data sources, which results in their low accuracy.

**Methods:**

In this paper, we propose a novel method, called MultiSourcDSim, to calculate disease similarity by integrating multiple data sources, namely, gene-disease associations, GO biological process-disease associations and symptom-disease associations. Firstly, we establish three disease similarity networks according to the three disease-related data sources respectively. Secondly, the representation of each node is obtained by integrating the three small disease similarity networks. In the end, the learned representations are applied to calculate the similarity between diseases.

**Results:**

Our approach shows the best performance compared to the other three popular methods. Besides, the similarity network built by MultiSourcDSim suggests that our method can also uncover the latent relationships between diseases.

**Conclusions:**

MultiSourcDSim is an efficient approach to predict similarity between diseases.

## Background

Quantitative measurement of disease similarity is gaining more and more attentions because it helps to reveal common psychophysiology and improve clinical decision-making systems, so as to better understand human diseases status and more accurately classify diseases [1]. It also plays a crucial role in identifying novel drug indications [2], since diseases may have the same or similar therapeutic targets, suggesting that they may be treated with the same or similar drugs [3–6]. In the past few decades, our understanding of human diseases has made remarkable progress [7]. For example, the network-based approaches [8–11] to calculating the similarity between diseases is impressive. Constructing a disease similarity network based on biological data to explore the relationship between diseases has become one of the research hotspots of modern biology and medicine. At present, the measurement of similarity disease research is necessary.

In previous studies, various properties of human genes (such as predicted function or amino-acid sequence length) and Gene Ontology (GO) [12–14] biological processes have been correlated with the chance of causing a disease [15–17]. The calculation approaches of disease similarity can be roughly divided into function-based methods [18, 19] and semantic-based methods [20]. The functional-based approach calculates similarities between diseases by comparing genes associated with diseases [18, 19]. For instance, the BOG [18] method, which was designed by Mathur and Dinakarpandian, calculates the similarity between diseases by comparing gene overlaps of related diseases. Moreover, BOG [18] also considers the self-information of each disease. However, its shortcoming is that it does not consider the functional link between disease-related genes. Further, Mathur and Dinakarpandian proposed a method based on process similarity (PSB [19]). The method provides functions to measure similarity, including the similarity function based on GO terms [12], and the similarity function between entities annotated with terms extracted from the ontology based on both co-occurrence and information content. The semantic-based method is extensively used in biomedical and bioinformatics. For instance, Resnik’s method [21] calculates the similarity between diseases according to the information content of the most informative common ancestor. Lin’s method [22] incorporates not only the information content of the most informative common ancestor but also the the information content of the two disease terms. Jiang and Conrath et al. [23] represented the similarity between two terms through the semantic distance.

In addition, phenotype similarity plays an important part in a lot of biological similarity and biomedical applications, and it is also the most common way of classifying diseases [24]. For example, the Human phenotype ontology (HPO) is a controlled and standardized vocabulary that describes the abnormal phenotype of human disease. And Medical Subject Headings (MeSH) [25] use this approach to classify diseases.

Although there are many patterns for measuring similarity between diseases, most of them use a single biological data source, and few methods using multiple biological data sources are proposed. For example, some of the previous approaches calculate the similarity according to genes related with diseases. Nevertheless, there exist some diseases which are unrelated or rarely related to genes. Thus, depending solely on individual biological data associated with disease might greatly affects the prediction performance of the methods. In this work, a novel approach named MultiSourcDSim is proposed to compute the similarity between diseases by integrating multiple biological datasets. In MultiSourcDSim, firstly, three disease similarity networks are respectively built by using a variety of biological data such as gene-disease associations, GO biological process-disease associations and symptom-disease associations. Secondly, the high-dimensional vector of each node is extracted by running restart random walks [26] on each network, and low-dimensional vectors that can represent the high-dimensional topological patterns in each network are learned. Finally, the similarity between diseases is obtained by calculating the cosine score between two low-dimensional vectors. The experiments demonstrate that disease similarity predicted by our method is significantly correlated with disease category of MeSH, implying that the network constructed by our method is capable of detecting the latent relationships between diseases. Moreover, the results also show that MultiSourcDSim outperform the other three popular methods.

## Methods

### Datasets

CTD’s MEDIC disease vocabulary which is downloaded in http://ctdbase.org (March 4, 2018) is chosen as criterion for describing diseases. CTD’s MEDIC disease vocabulary is a modified subset of descriptors from the Diseases [C] branch of the U.S. National Library of Medicine’s MeSH, combined with genetic disorders from the Online Mendelian Inheritance in Man (OMIM) database, and we use MeSH to mark disease terms. Each record in CTD’s MEDIC disease vocabulary contains 9 fields, 4 of which are retained for calculating disease similarity. They are respectively DiseaseID, DiseaseName, AltDiseaseIDs (alternative identifiers) and ParentIDs (identifiers of the parent terms).

We have collected three data sets associated with disease, namely gene-disease associations, GO biological process-disease associations, and symptom-disease associations. In the three sets, a great deal of biological information bound up with diseases is included. For instance, each record in the gene-disease associations contains 9 fields (GeneSymbol, GeneID, DiseaseName, DiseaseID, DirectEvidence, InferenceChemicalName, InferenceScore, OmimIDs, PubMedIDs). In the three data sets, 3,125,954 gene-disease associations containing 3254 disease terms and 668,760 GO biological process-disease associations containing 5720 disease terms are pooled from http://ctdbase.org(March 4, 2018), and each record in the two data sets is identified by MeSH markers. The gene terms and the gene ontology biological process terms are labeled with the NCBI gene identifiers and GO identifiers, respectively. The 80,638 symptom-disease associations are collected from paper [27], which describes 4040 diseases. However, the diseases in the symptom-disease associations are marked by the MeSH names. To obtain the Mesh identifiers corresponding to the names, we map the disease names in the symptom-disease associations to the IDs in the CTD’s MEDIC disease vocabulary. After screening for the co-occurring diseases term in all associations, 8126 diseases are extracted.

### Overview of MultiSourcDSim

In our method, we combine three disease-related data sets to calculate the similarity between diseases more accurately. Specifically, we firstly construct three disease similarity networks through computing the similarity respectively according to the gene-disease associations, GO biological process-disease associations, and symptom-disease associations. Secondly, the compact low-dimensional feature representations of diseases from the three similarity networks are learned by running Diffusion Component Analysis (DCA) [28–30]. Finally, the disease similarity is calculated according to the learned representations.

### Calculate semantic similarity of diseases

MeSH is a vocabulary that gives uniformity and consistency to the indexing and cataloging of biomedical literature. It is organized in a manner of tree structures with 16 main branches. Category C represents diseases. In our approach, the semantic similarity of diseases is measured by using the special structure between MeSH descriptor [25]. We build a directed acyclic graph (DAG) to clarify the associations among various diseases. The nodes in the DAG represent the MeSH descriptor. Child nodes are more specialized (containing more disease information) and parent nodes are more generalized (containing less disease information). In addition to the relationships of the disease itself, we also combine the relationships between disease and other biological entity, namely gene, GO and symptom. The probability of a disease occurs in a disease-related data set is just its frequency in the data set. The frequency of a disease term *t* is calculated as:

1 $$ f(t)=self(t)+\sum_{tc\in children(t)}f(tc).  $$

Here, *self*(*t*) represents the number of occurrences of the disease term *t* in a single data set, and the disease term *tc* is a direct child of the disease item *t*, belonging to the *children*(*t*) collection. In other words, the frequency of the disease term *t* in a single disease-related data set is defined as the frequency of its own occurrence plus the frequency of occurrence of all its child nodes. The probability that the disease term *t* appears in the disease-related data set is as follows:

2 $$ prob(t)=\frac{f(t)}{N}.  $$

Here, *N* indicates the frequency of occurrence of the root node in the corresponding DAG.

Then, the similarity scores are computed according to the probabilities of diseases based on the metric proposed by Lin et al. [22]. In Lin’s method, the similarity is measured in terms of information theory. It is believed that the similarity between terms is determined by their generality (information content of common ancestor nodes) and particularity (their respective information content). Therefore, the semantic similarity depends on the maximum ratio of the information content of the common ancestor nodes of the two terms to the sum of the information content of the two terms themselves. Generally, the higher the degree of information sharing between two terms, the higher the semantic similarity score, and on the contrary, the lower the similarity score. This definition is as follows:

3 $$ {\begin{aligned} Score(t1,t2)=\max_{t\in\left(LCA(t1,t2)\right)}\left(\frac{2 * \log prob(t)}{\log prob(t1)+ \log prob(t2)}\right). \end{aligned}}  $$

Here, *LCA*(*t*1,*t*2) is the set of least common ancestors of term *t*1 and *t*2. The similarity scores fall in the range [0, 1].

### Integrate multiple networks and learn representations

We construct three disease similarity networks according to the similarity scores. To achieve the compact integration of multiple similarity network, we adopt DCA strategy to capture low-dimensional vectors representing topological patterns of networks. In DCA, the random walk with restart (RWR) method [26] is firstly employed to analyze the structure of each network.

The RWR from a node *i* is defined as:

4 $$ s_{i}^{t+1}=(1-a)s_{i}^{t}T+ae_{i}.  $$

Here, *T* denotes the probability transfer matrix. $s_{i}^{t}$ is specified as an n-dimensional vector, where each entry is the probability of visiting a node at *t* iterations from the initial node *i*. *e*_*i*_ is the initial probability vector, where *e*_*i*_(*i*)=1 and *e*_*i*_(*j*)=0, ∀*j*≠*i*. *a* is the restart probability. After several iterations, a stable distribution is obtained, and *s*_*i*_ is regard as the ’diffusion state’ of the node *i*.

There exists noise in the diffusion states obtained in this manner, and the dimensionality is high. To solve this problem, we utilize fewer dimensions to approximate each diffusion state *s*_*i*_ through a polynomial logistic model based on the potential vector representation of nodes in a network. Specifically, the probability assigned to node *j* in the diffusion state of node *i* is as follows:

5 $$ \hat{s}_{ij}=\frac{exp{\left\{x_{i}^{T}w_{j}\right\}}}{\sum_{j'}exp{\left\{x_{i}^{T}w_{j}'\right\}}},  $$

where ∀*i*,*x*_*i*_,*w*_*j*_∈*R*^*d*^ for *d*≪*n*. *x*_*i*_ and *w*_*j*_ represent the node feature and context feature of node *i* respectively.

The goal is to find the low-dimensional vector representation of nodes *w* and *x* that best approximates a set of observed diffusion states *s*={*s*_1_,…,*s*_*n*_} according to the logistic model. To achieve the goal, KL-divergence is used as the objective function to optimize, which is given by:

6 $$ \mathop {\min }\limits_{w,x} C(s,\hat s) = \frac{1}{n}{\sum\nolimits}_{i = 1}^{n} {{D_{KL}}} \left({s_{i}}||{\hat s_{i}}\right),  $$

where *n* is the number of nodes. By writing out the definition of KL-divergence, the formula is written as:

7 $$ {\begin{aligned} \begin{array}{l} C\left(s,\hat s\right) =\\ \frac{1}{n}{\sum\nolimits}_{i = 1}^{n} { \left[ - H({s_{i}}) - \sum\limits_{j = 1}^{n} {{s_{ij}}} \left(x_{i}^{T}{w_{j}} - \log \left(\sum\limits_{j' = 1}^{n} {\exp \left\{ x_{i}^{T}{w_{j'}}\right\}}\right)\right)\right]}, \end{array} \end{aligned}}  $$

where *H*(·) denotes the entropy. In order to combine the three disease similarity networks, the formula (6) is modified as follows:

8 $$ \mathop {\min }\limits_{w,x} C(s,\hat s) = \frac{1}{n}{\sum\nolimits}_{m = 1}^{M} {{\sum\nolimits}_{i = 1}^{n} {{D_{KL}}} \left(s_{i}^{m}||\hat s_{i}^{m}\right)}.  $$

Here, *M* represents the number of networks. In this work, *M* is equal to 3. To minimize the objective function, we compute the gradients with regard to the parameters *w* and *x*. The low-dimensional vector representations are obtained by the quasi-Newton L-BFGS method with these gradients.

To improve efficiency, we can employ singular value decomposition (SVD) to optimize the alternative objective function [31].

### Calculate the similarity between diseases

After extracting the low-dimensional representations for all nodes which can best explain the connectivity patterns in the networks, we utilize the learned representations as features for calculating the disease similarity. In this study, the number of nodes in the three networks, namely the total number of diseases is 8126, and the dimension of these features is set to 600. The similarity between diseases is measured through cosine score, which is as follows:

9 $$ cosine(d_{x},d_{y})=\frac{\sum_{i}d_{x,i}d_{y,i}}{\sqrt{\sum_{i}d_{x,i}^{2}d_{y,i}^{2}}}.  $$

Here, *d*_*x*_ and *d*_*y*_ are two vectors which represent two disease respectively. Obviously, the similarity is between 0 and 1.

## Results

### The degree distribution of disease similarity networks

**Fig. 1:**
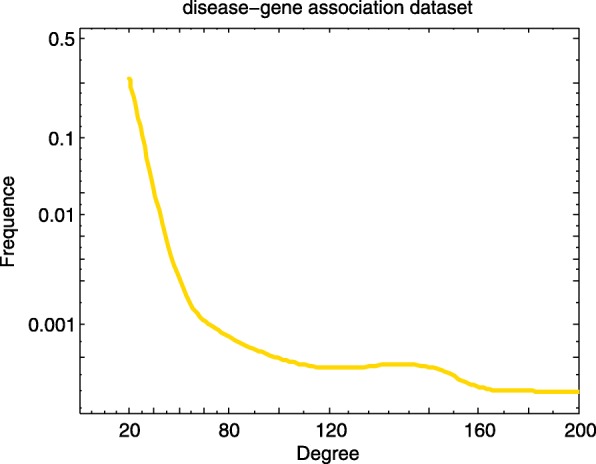
Degree distribution of disease node in the small similarity network built based on disease-gene association dataset

**Fig. 2:**
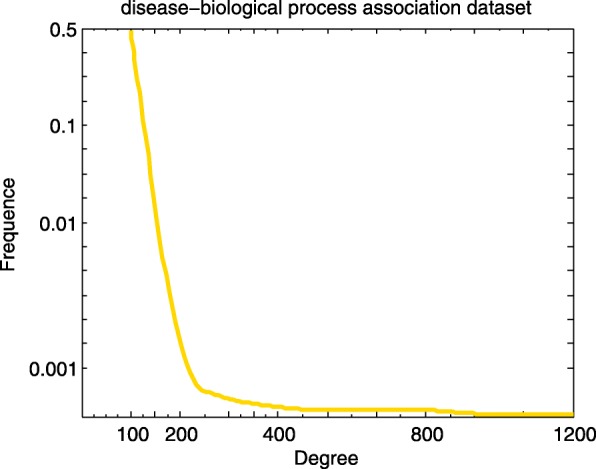
Degree distribution of disease node in the small similarity network constructed based on GO biological process-disease association dataset

**Fig. 3:**
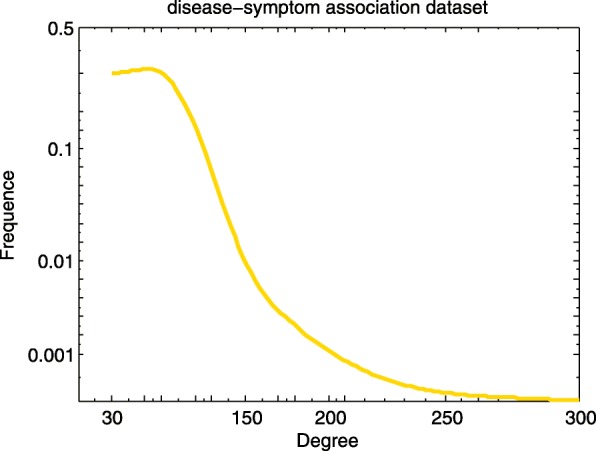
Degree distribution of disease node in the small similarity network constructed based on disease-symptom association dataset

We adopt gene-disease associations, GO biological process-disease associations and symptom-disease associations as the sources of disease similarity network, and construct the small similarity networks based on the Lin’s measure separately. In order to better understand the topology of these networks, we calculate the degree distribution of nodes in the network. Figures 1, 2 and 3 elucidates the degree distribution of disease node in three small disease similarity networks.

In the disease similarity network based on gene-disease association dataset (GDN), there exist 3254 diseases and 32733 connections. Marfan Syndrome (MeSH: D008382), which is the relation with 178 diseases, has the maximum degree. There are 225 diseases with degree 1 (Fig. 1). 5720 diseases and 249490 relationships make up the disease similarity network based on GO biological process-disease association dataset (BPDN). The disease with the maximum degree is Martin-Probst Deafness-Mental Retardation Syndrome (MeSH: C564495), the degree is 1024. As shown in Fig. 2, nearly half of the disease nodes have margins with about 100 other disease nodes. And similarity values of all disease pairs are computed in the disease similarity network based on symptom-disease association dataset (SDN), and the distribution of 48279 similarity values (between 4040 diseases) is acquired. Oculocerebrorenal Syndrome (MeSH: D009800) associated with 256 diseases has the maximum degree (Fig. 3). From the above calculation we can draw a conclusion that the density of GDN is the largest compared to BPDN and SDN.

**Fig. 4:**
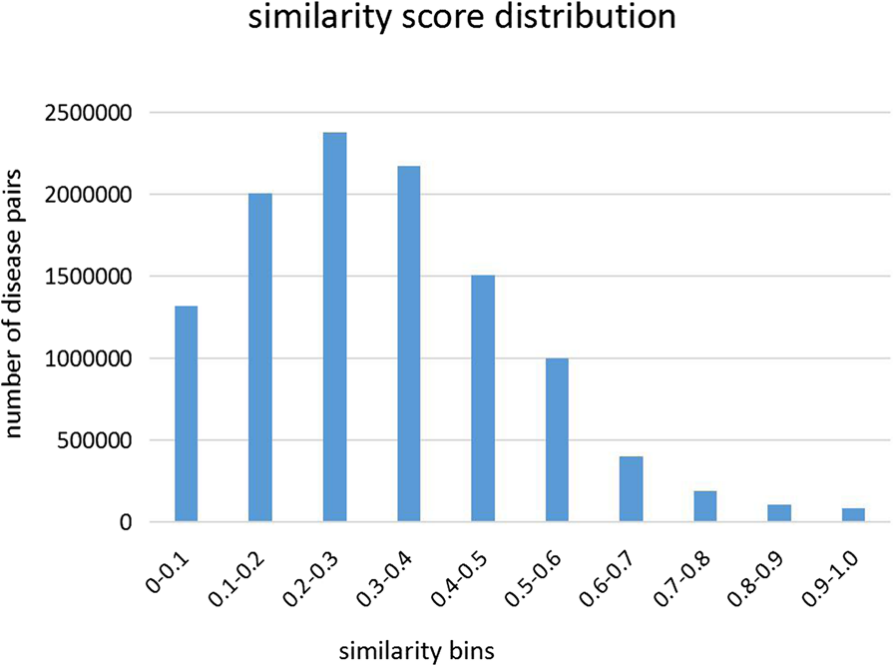
Histogram of similarity scores between 8126 disease nodes. Most disease-disease pairs have a low similarity score

After obtaining the integrated disease similarity network (GPSN), the distribution of these similarity scores are also counted. The distribution is represented in Fig. 4, the similarity scores for most disease pairs across the network ranges from 0 to 0.6. The number of disease pairs in the 0.2-0.3 similarity bin is the highest, followed by the 0.3-0.4 bin.

### Benchmark

The benchmark set which is adopted in this experiment contains 40 pairs of highly similar diseases. It is derived from the work of Suthram et al. [1] and Pakhomov et al. [32], and cancers are deleted. The benchmark set consists of pairs of diseases that are confirmed to be interrelated, such as Polycystic Ovary Syndrome(MeSH: D011085) and Obesity(MeSH: D009765), Chronic Obstructive Airway Disease(MeSH: D029424) and Asthma (MeSH: D001249). It also contains some diseases pairs which have no apparent correlations, but have proved to be correlated through various evidences, such as Obesity and Asthma, Malaria (MeSH: D008288) and Anemia (MeSH: D000740). Moreover, we randomly choose 500 disease pairs from the similarity network as a random set, where the disease pairs in the benchmark set are deleted.

### Parameter selection

**Fig. 5:**
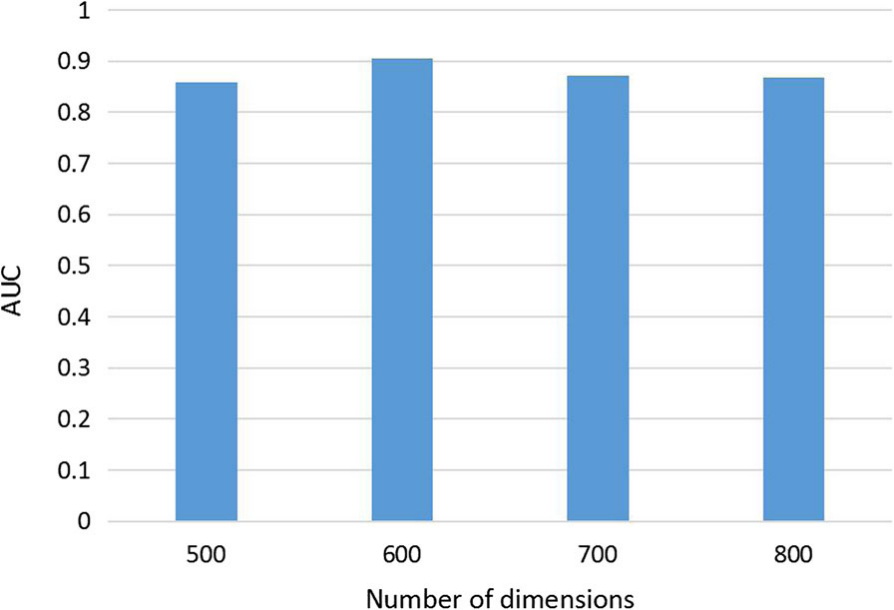
Comparision for different numbers of dimensions

There are two parameters (*α* and *d*) to be tuned in MultiSourcDSim. The parameter *α* is the restart probability. According to previous practical experience [33], it is set to 0.5. The parameter *d* denotes the feature dimension of each node. We compare the performance for different numbers of dimensions based on the benchmark set. We calculate the values of AUC when *d* is increasing from 500 to 800 with step size 100. As shown in Fig. 5, the results show that the performance of MultiSourcDSim is stable over a wide range of values for the number of dimensions, implying that our method is robust to over-fitting. On the whole, the AUC comes to the max value when *d* equals 600. Hence, *d* is set to 600 in this paper.

### Performances assessment

**Fig. 6:**
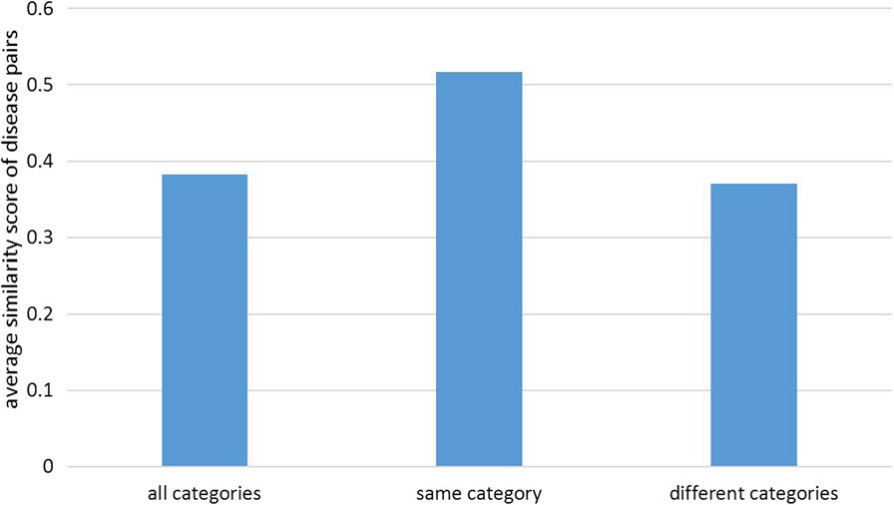
Evaluation of MultiSourcDSim against MeSH classification

To evaluate the disease similarity results calculated by MultiSourcDSim, we make a comparison on the disease classification of MeSH. MeSH is an authoritative medical thesaurus and the basis for biomedical indexing. MeSH divides the disease (C) sections into 26 categories according to the tree code (excluding some ambiguous categories). To discuss whether GPSN is related to the MeSH disease category, we examine the difference between the similarity scores of disease pairs belonging to the same MeSH category and the similarity scores of disease pairs of different MeSH categories. As demonstrated in Fig. 6, the average similarity scores for disease pairs from the same MeSH are significantly higher than those from different MeSH categories. In conclusion, the experiment demonstrates that the similarity scores of disease pairs are closely relevant to MeSH disease category.

**Fig. 7:**
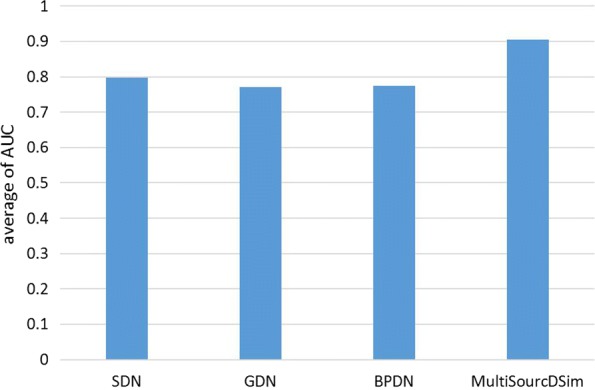
Integrating Multiple Networks Outperforms Individual Networks

Moreover, in order to verify that the performance of the network integrating the three data sets is better than that of the network formed by the single data set, we compare GDN, BPDN, SDN and GPSN based on the banchmark set and random set. AS shown in Fig. 7, MultiSourcDSim achieves the best AUC of 0.906, and the AUC values of GDN, BPDN and SDN are 0.771, 0.774 and 0.797, respectively. This result indicates that compared to individual networks without integration, MultiSourcDSim has a more stable and stronger power for discovering disease-disease associations. The performance improvement is partially attributed to the fact that synthetical analyzing the structure of the the multiple networks can uncover fine-grained topological patterns. Another important factor is the compactness of the feature representations, which help capture the relevant topological patterns apart from noise in the data.

The performance of MultiSourcDSim is further evaluated by comparing it with other three recent approaches: the text-based approach, namely MimMiner [34], an integrated semantic and functional approach, called MedNetSim [35], and the web-based approach, HSDN [27].

To fairly compare the performance of these methods, we select widely used metrics, such as accuracy (ACC), the area under the ROC curve (AUC), F1-score (F1), the Matthew’s correlation coefficient (MCC), precision (PRE), sensitivity (SEN/Recall) and specificity (SPE). Based on the four approaches, we compute the the similarity scores of disease pairs in the benchmark set and the random set, and sort them in descending order, respectively. Moreover, we look on the disease pairs in the benchmark set and the random set as positive and negative samples, respectively. The disease pairs correctly predicted in the benchmark set are considered to be true positive samples, and the disease pairs in the random set which are predicted to be highly correlated are thought of as false positive samples. The results of the evaluation are shown in Table 1, where the AUC value of the HSDN method is the minimum, which is 0.818. The MimMiner method applies text mining to disease classification and improves performance, resulting in an AUC of 0.836. The MedNetSim method takes the entire protein interactions and the biomedical literature corpus into consideration, increasing its AUC to 0.854. Our approach integrates multiple disease-related data sets and further improves the performance with an AUC value of 0.905, which is the best in the four methods. In addition, our method also achieves the highest values for ACC, F1, MCC, PRE, and SEN, which are 0.815, 0.684, 0.273, 0.601, and 0.750, separately.

**Table 1:** Prediction performance of MultiSourcDsim in comparison with other three methods on the benchmark set and random set

Methods	ACC	AUC	F1	MCC	PRE	SEN	SPE
MultiSourcDSim	**0.815**	**0.905**	**0.684**	**0.273**	**0.601**	**0.750**	0.656
HSDN	0.688	0.818	0.409	0.263	0.375	0.450	0.750
MimMiner	0.652	0.836	0.400	0.259	0.334	0.500	**0.875**
MedNetSim	0.630	0.854	0.391	0.224	0.361	0.425	0.874

The black bold fonts represent the optimal value

**Fig. 8:**
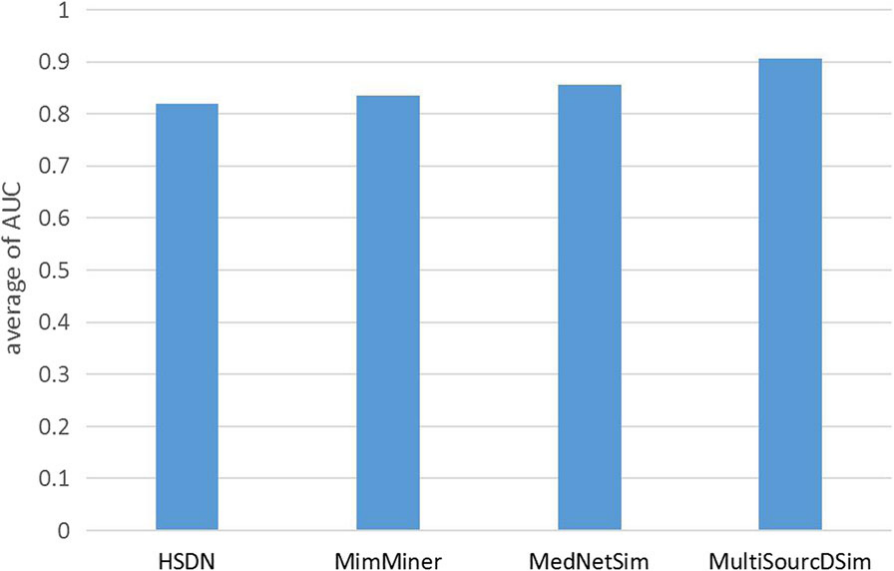
Average of AUC for 100 permutations

**Fig. 9:**
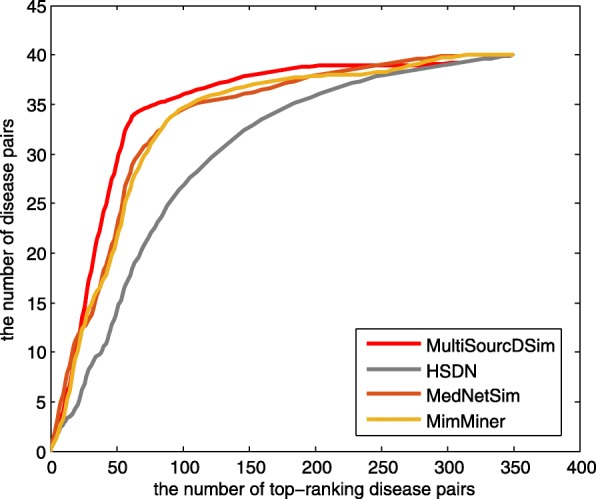
The number of disease-pairs with varying the number of top-ranking disease pairs

The results in Table 1 demonstrate that calculating disease similarity by integrating multiple disease-related data sources is an effective method. In order to test the stability of our method, we randomly select 100 disease pairs and compute their similarity scores. The calculations are repeated 100 times and the average AUC of the four methods are depicted in Fig. 8. The average values are respectively 0.819 (HSDN), 0.835 (MimMiner), 0.855 (MedNetSim) and 0.906 (MultiSourcDsim), which are consistent with the AUC column in Table 1. We further compare the ranking of disease pairs derived from the benchmark set. As shown in Fig. 9, The number of the solution disease pairs which are found by MultiSourcDsim always are the largest in the top 220 disease pairs.

In addition, by using the lowest ranked disease pairs in 540 disease pairs (500 random disease pairs and 40 benchmark pairs), MultiSourcDSim can find all 40 benchmark pairs, which represents quite good performance. For example, Obesity (MeSH: D009765) and Asthma (MeSH: D001249) are disease pairs belonging to the benchmark set, which ranks last in our approach. As shown in Table 2, the average ranking of Obesity and Asthma is very low among all the four methods. Nevertheless, compared to the other three methods, our approach has increased the ranking of Obesity and Asthma by 9%-14%.

**Table 2:** The average ranking of the disease pair (Obesity and Asthma) in 540 disease pairs

	HSDN	MimMiner	MedNetSim	MultiSourcDSim
average ranking	252.5	257.4	242.9	**220.6**

### Integrated disease similarity network

**Fig. 10:**
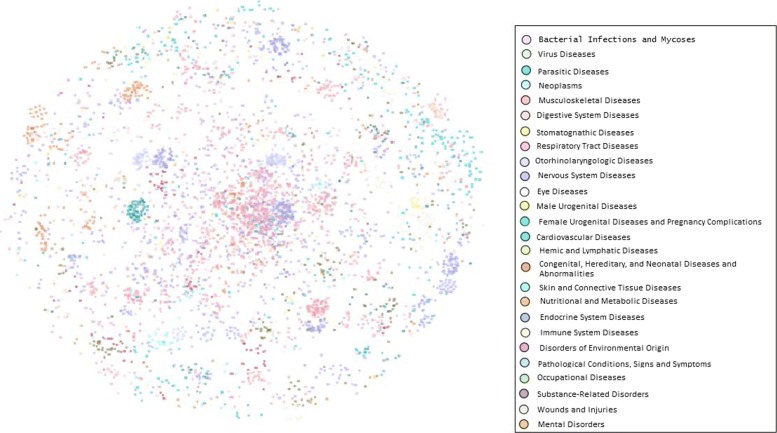
An overview of disease similarity network (GPSN) based on our method results. Nodes were coloured according to the MeSH category to which they belong

**Fig. 11:**
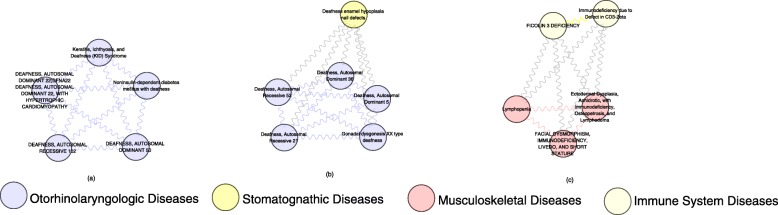
Three connected components from the disease similarity network constructed by our method

We construct a disease similarity network by using the top-ranking 0.3% of the similarity values in 8126 diseases. As shown in Fig. 10, there are 2604 diseases in the network and they are connected to each other by 121787 edges. The maximum connected component consists of 283 nodes. Martin-Probst Deafness-Mental Retardation Syndrome (MeSH: C564495), which is connected to 511 diseases, has the maximum degree. In Fig. 10, nodes in the network represent diseases, and the nodes are colored different colors. Each color is corresponding to a different MeSH category, such as Virus Diseases (MeSH: C02), Digestive System Diseases (MeSH: C06), Eye Diseases (MeSH: C11), Immune System Diseases (MeSH: C20) and so on. For each classification, diseases in the same MeSH category are usually similar to each other, such as disease of Musculoskeletal Diseases (MeSH: C05) category, disease of Nervous System Diseases (MeSH: C10) category, and so on. Figure 11 also shows the feature that diseases within one class are more probable to gather in the same neighbourhood with each other. For instance, 5 diseases belonging to the Otorhinolaryngologic Diseases classification constitute a small component. As shown in the Fig. 11a, all of these 5 diseases are deafness. Six diseases generate another connected component (Fig. 11b), five of which are Otorhinolaryngologic Diseases and the other is Stomatognathic Diseases. These demonstrations further indicate that the similarity scores of disease pairs belonging to the same category in the results computed by MultiSourcDSim are greater than those between belonging to different categories.

Besides identifying relationships between diseases belonging to the same disease classification, our approach can also find the associations beween diseases belonging to different classifications. For instance, as shown in Fig. 11c, three Musculoskeletal Diseases are linked to two Immune System Diseases by our method. Among the three Musculoskeletal Diseases, it has been reported that people with Lymphopenia might have immune system diseases.

## Discussion and conclusion

Determining the correlation between diseases helps to deepen understanding of the potential mechanisms among diseases. There are many studies about the association between diseases, such as predicting disease-related genes [36–38] and new drug indications [2]. In addition, a huge challenge for researchers in modern biology [39, 40] is how to get more information about the disease. In the past few decades, many researchers have proposed a number of methods to predict the similarity between diseases (for example, build a network of disease similarity) based on biological data and make a great progress. However, these methods use only a single biological data and do not consider combining multiple biological data as a basis for predicting disease similarity.

In this paper, we propose a novel method, MultiSourcDSim, to predict similarity between diseases, which builds a disease similarity network based on multi-faceted biological data related to disease. According to the similarity scores computed by our method, we can conclude that the similarity scores of disease pairs belonging to the same MeSH classification are significantly higher than those of disease pairs belonging to different MeSH classifications. And, comparing the performance of the MultiSourcDSim method with the other three methods (MimMiner [34], MedNetSim [35] and HSDN [27]) under the same benchmark set, we have found that our method is superior. Furthermore, the disease similarity network constructed by our method can also uncover latent relationships between diseases.

Although multiple disease-related data sources are integrated to compute similarities between diseases, there may be some bias due to incomplete data. In addition to considering the integration of multiple biological data, we also need to take into account the modular nature of each disease in further study of the similarities between diseases, since the modularity of each disease module can give more information [41–43]. Moreover, disease networks have proven useful for predicting novel therapeutic applications of known compounds [44] and inferring novel disease genes [45].

## Data Availability

The datasets used in this study is available at http://ctdbase.org.

## Abbreviations

ACC: Accuracy
AUC: The area under the ROC curve
BPDN: GO biological process-disease association network
CTD: The comparative toxicogenomics database
DAG: Directed acyclic graph
DCA: Diffusion component analysis
F1: F1 score
GDN: Gene-disease association network
GO: Gene ontology
GPSN: The integrated disease similarity network
HPO: Human phenotype ontology
MCC: The Matthew’s correlation coefficient
MeSH: Medical subject headings
NCBI: National center for biotechnology information
OMIM: Online mendelian inheritance in man
PRE: Precision
RWR: Random walk with restart
SDN: Symptom-disease association network
SPE: Specificity
